# The effect of oral protein and carbohydrate solution administration on NLR, IL-6 and CRP levels in patients undergoing surgery

**DOI:** 10.62838/jccm-2026-0011

**Published:** 2026-04-30

**Authors:** Mahendratama P. Adhi, Oscar Febriano, Alfi Yasmina, Iwan Nuryawan, Agung Ary Wibowo, Kenanga Marwan Sikumbang

**Affiliations:** Department of Anesthesiology, Faculty of Medicine and Health Sciences, Lambung Mangkurat University, Banjarmasin, Indonesia; Department of Pharmacology, Faculty of Medicine and Health Sciences, Lambung Mangkurat University,Indonesia; Department of Surgery, Digestive Division, Faculty of Medicine and Health Sciences, Lambung Mangkurat University,Indonesia

**Keywords:** protein and carbohydrate solution, neutrophil-lymphocyte ratio, interleukin-6, C-reactive protein

## Abstract

**Aims:**

To determine the effect of administering oral protein and carbohydrate solutions the C-Reactive Protein (CRP), Interleukin-6 (IL-6), and Neutrophil-Lymphocyte Ratio (NLR) in patients planned any surgery

**Methods:**

A double-blind, randomized clinical study, at Ulin Regional Hospital, Banjarmasin with patients planned any surgery. This research had 80 patients in total (40 subjects in the control group and 40 subjects in the intervention group). Before surgery, 200 mL of a protein and carbohydrate solution per oral was given to the intervention group, while a placebo was given to the control group. Twenty-four hours after surgery, each subject's levels of CRP, IL-6 and NLR were measured. Statistical Package for the Social Sciences Version 29 was used to analyze the data.

**Results:**

NLR at 24 hours postoperatively in the intervention group was lower than in the control group, but not statistically different (8.65±4.33 vs. 7.86±4.65, p=0.308). The IL-6 level at 24 hours postoperatively in the intervention group was significantly lower than in the control group (9.49 (6.03–22.65) vs. 20.08 (11.64–50.11), p=0.011). Although not statistically different, the CRP level at 24 hours postoperatively in the intervention group was lower than in the control group (15.10 (7.20–41.60) vs. 34.70 (11.87–71.55), p=0.056). There was no difference in postoperative nausea or vomiting between the two groups.

**Conclusion:**

Postoperative interleukin-6 levels have been demonstrated to decrease when oral protein and carbohydrate solutions are given to patients undergoing surgery; however, NLR and CRP levels have not been seen to decrease.

## Introduction

Every surgical procedure requires anesthesia. General anesthesia is one kind of anesthesia. Sedation, forgetfulness, analgesia, and immobility are all side effects of general anesthesia, a drug-induced, reversible condition that preserves physiological stability [1]. Pro-inflammatory cytokines may be impacted by different anesthetics. Pro-inflammatory cytokines including C-reactive protein (CRP) and interleukin-6 (IL-6) have been shown to be inhibited by propofol [2]. However, volatile anesthetics have both immunosuppressive and immunoactivating effects. For example, sevoflurane reduces NK cell cytotoxicity, cytokine release, and neutrophil cell count and adhesion [3].

On the other hand, fasting before surgery can increase the surgical stress response [4]. Enhanced Recovery After Surgery (ERAS) currently recommends oral carbohydrate administration 2–3 hours before surgery, typically an isotonic complex carbohydrate solution with or without protein. Preoperative energy reserves are necessary to support stress-induced reserve mobilization, ensuring that physiological integrity and strength are not compromised [5]. Research by Ravanini et al. (2015) showed that administering a combination of protein and carbohydrate solution 2 hours before surgery resulted in lower insulin resistance and therefore could reduce the organic response to trauma [6].

Research by Haider et al. (2023) also showed that consuming a carbohydrate-rich beverage reduced the inflammatory response associated with surgical trauma in the pre- and postoperative periods. This was evidenced by lower CRP levels compared to the group that did not receive the carbohydrate-rich beverage [7]. According to Rizvanovic et al. (2023), giving a mix of protein and carbohydrate solutions before surgery decreased the frequency and severity of postoperative problems as well as postoperative Neutrophil-Lymphocyte Ratio (NLR) values [8]. Research related to the effects of administering a combination of carbohydrate and protein solutions on NLR, IL-6 and CRP is still limited, and more studies have examined oral carbohydrate solutions before pre-operative compared to combination solutions with protein. The purpose of this study was to verify the effect of protein and carbohydrate solutions for NLR, CRP, and IL-6 levels in patients planned any surgery.

## Methods

### Study Design

This study is a randomized controlled trial (RCT), double blind, in Ulin Regional Hospital, Banjarmasin, South Kalimantan

### Participants

The minimum sample size is calculated using the unpaired analysis formula with a total of 40 subjects for each group. All of the study's participants were patients planned any surgery. Patients who were ≥ 18–69 years old, American Society of Anesthesiologists (ASA) I and II, had a body mass index (BMI) between 18.5 and 30 kg/m^2^, were undergoing elective surgery, had surgical complexity levels 2 and 3, and were willing to sign an informed consent form were all eligible to participate in this study. Patients with diabetes mellitus (HbA1c ≥ 6.5%), patients with comorbidities such as kidney disorders (urea > 100 mg/dL and creatinine > 5 mg/dL), impaired liver function (increased SGOT/SGPT > 5x normal value), and patients who were required to fast for 24 hours post-surgery were excluded from this study. Dropout criteria for this study were patients who underwent reoperation < 24 hours after the first operation, patients with endotracheal tube retention and ICU care, patients who failed blood draws.

### Randomization

The research sample was taken using consecutive sampling technique with a randomization process using a closed envelope technique which determined the grouping of subjects into a control group or an intervention group.

### Intervention

The intervention group was given a protein and carbohydrate combination solution using Fresubin Jucy^®^ (Fresenius Kabi, Germany), which was poured into an empty 200 ml bottle wrapped in black plastic. One hundred ml of fresubin juice contains 150 kcal, 4.0 g protein, 33.5 g carbohydrates, osmolarity 680 mosmol/l, contains various minerals such as sodium, potassium, chloride, calcium, magnesium, phosphorus, iron, zinc, copper, manganese, iodine, fluoride, chromium, molybdenum, and selenium; as well as various vitamins such as vitamin A, beta carotene, D3, E, K1, B1, B2, niacin, B6, B12, pantothenic acid, biotin, folic acid, and vitamin C. The control group was given a placebo solution of Pristine^®^(Super Wahana Tehno, Indonesia) brand mineral water, poured into an empty 200 ml bottle wrapped in black plastic. Pristine^®^ containing pH 8.6+. The solution was given 3 hours before surgery and had to be consumed within 15 minutes per oral, controlled by the research team. This study was double-blinded. Both solutions (Fresubin Jucy^®^ and Pristine^®^) were wrapped in black plastic to blind the subjects and the researchers analyzing the study. A key code was then placed on each black plastic. The key code was held by another member of the implementation team.

### End Point and Follow-Up

NLR, IL-6, and CRP were measured before and 24 hours postoperatively in all study subjects. PONV was also assessed in each study subject. IL-6 and CRP were examined using radioimmunoassay. Neutrophil and lymphocyte counts were examined using a hematology analyzer to assess the NLR.

### Anesthesia Procedure

All study subjects received the same anesthetic procedure.

The patient underwent general anesthesia via endotracheal intubation using the following medications: Fentanyl (Mahakam Beta Farma, Indonesia) at an initial dose of 2–3 mcg/kg/iv, propofol (Fresenius Kabi, Indonesia) 2–3 mg/kg/iv, and atracurium (Ethika Industri Farmasi Company, Indonesia) 0.5 mg/kg/iv to facilitate intubation. This was followed by administration of fentanyl via syringe pump at a dose of 0.5–1 mcg/kg/hour/iv. Maintenance was provided during surgery with sevoflurane (Pratapa Nirmala Company, Indonesia) 1.5–2 vol% and oxygen FiO2 50%.

After the patient was intubated, the operation was performed.

The patient was maintained under the triad of anesthesia (sedation, analgesia, and relaxation) and maintained normovolemia, normotension, and normothermia.

After the operation was completed, the fentanyl injection via syringe pump was discontinued.

Postoperative analgesia was administered according to the level of postoperative pain. In operations with a mild pain scale, the analgesic ketorolac (Interbat, Indonesia) 30 mg is used and for operations with a moderate to severe pain scale, ketorolac 30 mg is used in combination with tramadol (MBK Mahakam, Indonesia) 100 mg intravenously.

### Data analysis

Statistical Product and Service Solution (SPSS) software version 29.0 was used to analyze the data. Subject characteristics included age, gender, BMI, surgical level, duration of surgery, and ASA status in the control group and the protein and carbohydrate combination solution group. Age, BMI, and duration of surgery are presented as means and standard deviations. Gender, surgical level, and ASA status are presented as percentages and frequencies. The Shapiro-Wilk test was used to determine whether age and BMI were normal. Differences in age and surgical duration between the two groups were assessed using the Mann-Whitney test. Differences in gender and surgical level between the two groups were assessed using the chi-square test. Differences in BMI between the two groups were determined using an independent t-test. To determine whether the ASA characteristics of the two groups differed, the Fisher exact test was used.

Data normality testing for NLR, IL-6, and CRP variables was also performed using the Shapiro-Wilk test. The results showed that the data were not normally distributed, therefore, the effect of protein and carbohydrate solution administration on NLR, IL-6, and CRP levels in the intervention group was analyzed. The 95% confidence interval was used. A p-value less than 0.05 is considered significant.

### Research Ethics

All research procedures were approved by parents, guardians, and patients through a consent form after they were informed of the planned procedures and this research was declared to meet ethical standards by the Health Research Ethics Commission, Faculty of Medicine, Lambung Mangkurat University (number: 17/PPDS.Ans/Litbang/RSUDU/III/2025). Before conducting the examinations required in this research, each research subject who agreed to this research procedure had to sign an informed consent.

## Results

Eighty patients were planned surgery at Ulin Regional Hospital in Banjarmasin participated in this trial; forty were assigned to the control group and forty were given a combination of protein and carbohydrate solutions. Unfortunately, one person dropped out of the group that received both protein and carbohydrate solutions, leaving 40 in the control group and 39 in the group that received both. The two groups did not differ significantly in terms of topic characteristics (Table 1).

**Table 1.** Patient characteristics

**Variable**	**Group**		**p-value**
	**Control (n=40)**	**Intervention (n=39)**	
Age (years), mean±SD	40.22±12.06	40.38±11.47	0.953*

Gender			
Men	15 (37.5%)	10 (25.6%)	0.257**
Women	25 (62.5%)	29 (74.4%)	
BMI (kg/m^2^), mean±SD	23.67±3.24	23.50±3.80	0.839***

Operation level			
Level 2	27 (67.5%)	20 (51.3%)	0.142**
Level 3	13 (32.5%)	19 (48.7%)	
Operation time (minutes)	144.25±61.75	161.41±72.99	0.383*

ASA Status			
ASA I	1 (2.5%)	1 (2.6%)	1.000****
ASA II	39 (97.5%)	38 (97.4%)	

Notes: BMI: Body Mass Index, ASA: American Society of Anesthesiologists, SD: Standard Deviation;

* Mann-Whitney,

** Chi-square,

*** Independent T-Test,

**** Fisher Exact

Table 2 shows an analysis of the effect of administering a combination of protein and carbohydrate solutions on NLR, IL-6, and CRP. NLR, IL-6, and CRP levels increased 24 hours postoperatively compared to preoperative levels, especially in the control group. However, these differences were not statistically significant (p=0.220, 0.096, and 0.062, respectively).

**Table 2.** The effect of administering a combined protein and carbohydrate solution on NLR, IL-6 and CRP

**Variable**	**Group**		**p-value**
	**Control (n=40)**	**Intervention (n=39)**	
NLR, mean±SD			
Pre-Operation	3.24±2.17	3.29±2.63	0.818
24 hours post operation	8.65±4.33	7.86±4.65	0.308
Δ Post-Pre Operative	5.51±3.70	4.57±4.60	0.220

IL-6 (pg/ml), median (IQR)			
Pre-Operation	4.08 (2.00–9.77)	2.88 (2.00–5.74)	0.110
24 hours post operation	20.08 (11.64–50.11)	9.49 (6.03–22.65)	**0.011**
Δ Post-Pre Operative	16.40 (3.00–38.02)	5.30 (2.56–17.33)	0.096

CRP (mg/dL), median (IQR)			
Pre-Operation	2.65 (1.12–13.12)	2.90 (1.30–7.20)	0.803
24 hours post operation	34.70 (11.87–71.55)	15.10 (7.20–41.60)	0.056
Δ Post-Pre Operative	26.40 (7.20–41.60)	9.90 (2.30–30.60)	0.062

Notes: IL-6: Interleukin-6, CRP: C-Reactive Protein, NLR: Neutrophil-Lymphocyte Ratio, SD: Standard Deviation, Δ: delta (mean change pre-operatively to post-operatively)

Table 3 shows the effect of administering a combination of protein and carbohydrate solutions on NLR, IL-6, and CRP at level 2 surgical complexity. The results showed an increase in NLR after 24 hours post-surgery compared to before surgery, especially in the control group. However, There was no statistically significant difference in the NLR rise between the two groups (p = 0.138). Nevertheless, the data also revealed that IL-6 and CRP had increased 24 hours after surgery as compared to before, particularly in the control group as opposed to the intervention group. This difference in rise was statistically significant (p = 0.035 and 0.024, respectively). In this study, there were several subjects who had post-operative CRP levels that were much lower than pre-operative CRP levels, resulting in a negative figure in the median (IQR) Δ Post-Pre Operative.

**Table 3.** The effect of protein and carbohydrate combination solution on NLR, IL-6, and CRP at level 2 surgical complexity

**Variable**	**Group**		**p-value**
	**Control (n=40)**	**Intervention (n=39)**	
NLR, mean±SD			
Pre-Operation	3.12±2.03	2.61±1.89	0.241
24 hours post operation	8.74±4.09	6.92±3.91	0.060
Δ Post-Pre Operative	5.77±3.38	4.30±4.54	0.138

IL-6 (pg/ml), median (IQR)			
Pre-Operation	4.37 (2.00–9.40)	2.64 (2.00–3.79)	**0.036**
24 hours post operation	19.00 (6.34–38.96)	7.00 (3.81–9.47)	**0.004**
Δ Post-Pre Operative	14.76 (1.98–26.52)	3.27 (1.54–5.22)	**0.035**

CRP (mg/dL), median (IQR)			
Pre-Operation	2.50 (1.00–7.50)	3.25 (0.90–5.02)	0.568
24 hours post operation	24.40 (9.90–45.80)	9.55 (3.65–14.52)	**0.015**
Δ Post-Pre Operative	16.90 (5.20–42.10)	4.45 (–0.07–10.65)	**0.024**

Notes: IL-6: Interleukin-6, CRP: C-Reactive Protein, NLR: Neutrophil-Lymphocyte Ratio, SD: Standard Deviation, Δ: delta (mean change pre-operatively to post-operatively

The impact of giving a mix of protein and carbohydrate solutions on NLR, IL-6, and CRP at level 3 surgical complexity is displayed in Table 4. NLR, IL-6, and CRP increased more in the control group than in the intervention group, but the difference was not statistically significant (p=0.954; 0.478 and 0.103, respectively)

**Table 4.** The effect of administering a combined protein and carbohydrate solution on NLR, IL-6 and CRP at level 3 surgical complexity

**Variable**	**Group**		**p-value**
	**Control (n=40)**	**Intervention (n=39)**	
NLR, mean±SD			
Pre-Operation	3.50±2.50	4.01±3.12	0.367
24 hours post operation	8.46±4.06	8.86±5.24	0.893
Δ Post-Pre Operative	4.96±4.38	4.85±4.77	0.954

IL-6 (pg/ml), median (IQR)			
Pre-Operation	3.79 (2.15–12.28)	3.62 (2.00–7.67)	0.603
24 hours post operation	27.02 (18.51–93.43)	22.56 (10.48–40.85)	0.199
Δ Post-Pre Operative	17.37 (12.17–83.70)	15.65 (5.81–27.86)	0.478

CRP (mg/dL), median (IQR)			
Pre-Operation	3.80 (1.75–33.95)	12.50 (1.30–33.60)	0.803
24 hours post operation	66.40 (34.90–84.90)	36.90 (24.40–68.50)	0.140
Δ Post-Pre Operative	41.00 (19.45–70.00)	25.80 (7.30–55.50)	0.103

Notes: IL-6: Interleukin-6, CRP: C-Reactive Protein, NLR: Neutrophil-Lymphocyte Ratio, SD: Standard Deviation, Δ: delta (mean change pre-operatively to post-operatively)

**Table 5.** Factors affecting changes in NLR, IL-6 and CRP in surgery patients

**Variable**	**NLR**			**IL6**			**CRP**		

	**p-value**	**Regression coefficient**	**95% (Lower-Upper)**	**p-value**	**K Regression coefficient**	**95% (Lower-Upper)**	**p-value**	**Regression coefficient**	**95% (Lower-Upper)**
Age	0.315	–0.41	–0.122–0.040	0.102	–0.710	–1.564–0.144	0.785	–0.099	–0.816–0.619
Administration of a combination of protein and carbohydrate solutions	0.233	–1.146	–3.045–0.752	0.072	–18.285	–38.273–1.704	**0.028**	–18.919	–35.709 – (–2.129)
Gender	0.859	0.187	–1.914–2.288	0.199	–14.370	–36.490–7.750	0.496	–6.376	–24.955 – 12.204
ASA	0.985	0.056	–5.989–6.101	0.797	8.231	–55.415–71.878	**0.002**	–86.232	–139.692 – (–32.772)
Operation Level	0.496	–0.718	–2.810–1.374	0.052	21.826	–0.201–43.853	**0.045**	18.906	0.404 – 37.407
Operatime Time	**0.037**	0.016	0.001–0.031	0.803	–0.020	–0.78–0.138	0.563	0.039	–0.094–0.72
BMI	0.166	–0.190	–0.461–0.081	0.344	–1.363	–4.213–1.488	0.356	–1.116	–3.511–1.278

Notes: NLR: Neutrophil-Lymphocyte Ratio, IL-6: Interleukin-6, CRP: C-Reactive Protein, ASA: American Society of Anesthesiologists, BMI: Body Mass Index

**Table 6.** PONV Incidence in the control group and the protein and carbohydrate combination solution group

**PONV**	**Control Group**	**Intervention Group**
No	40 (100%)	39 (100%)
Yes	0 (0%)	0 (0%)

Multivariate analysis was also performed in this study to assess factors influencing changes in NLR, IL-6, and CRP. Multivariate testing was performed using multiple linear regression.

This study assessed the incidence of PONV to determine the side effects of administering a combination protein and carbohydrate solution. However, no PONV events were reported in this study.

## Discussions

The purpose of this study was to analysis the effect of protein and carbohydrate solutions administrations affected NLR, IL-6 and CRP levels in patiens planned surgery. The findings indicated that the group that received the mix of protein and carbohydrate solutions had a smaller mean rise in NLR 24 hours after surgery than the control group, although not statistically significant. This negative outcome could suggest that NLR is not a suitable metric for evaluating post-operative inflammation. These findings contradict the research of Rizvanovic et al. (2023), which revealed that the control group's postoperative NLR and delta NLR values were noticeably higher (p < 0.001; p < 0.001) [8].

Postoperative alterations in leukocyte levels (leukocytosis, neutrophilia, and lymphopenia) are anticipated on the first day of treatment, resulting from the influence of endogenous cortisol, catecholamines, and cytokines in reaction to surgery, anesthesia, and hemorrhage [9,10]. The degree of the immuno-inflammatory reaction brought on by surgery is indicated by delta NLR. Several inflammatory cells are stimulated by cytokines produced during surgery. It has been established that IL-6 influences the differentiation of B cells. They will stimulate neutrophils, monocytes, macrophages, CD4 T lymphocytes by TNF-α and IL-8 which will result in cell adhesion, chemotaxis, recruitment, as well as microvascular disruption [8].

Researchers reported that preoperative administration of a protein and carbohydrate combination solution maintained preoperative HLA-DR expression levels on monocytes after surgery. Meanwhile, preoperative fasting reduced HLA-DR expression. Therefore, administering a protein and carbohydrate combination solution may lower NLR by maintaining HLA-DR expression levels [8]. Based on the multivariate analysis of this study, the duration of surgery affected changes in the mean NLR. The longer the surgery, the greater the increase in the NLR. As is known, the NLR increases within <6 hours after acute physiological stress and continues to increase until it begins to decline on the third postoperative day [9,10]

The study's findings also revealed an interesting difference in IL-6 levels 24 hours after surgery between the control group and the group that received a protein and carbohydrate solution. This result is consistent with a research by Hu et al. (2021) that found a substantial difference in IL-6 levels between the group that received a carbohydrate solution and the control group [11]. Tissue damage from surgery sets off a stress reaction. TNF-α, IL-6, IL-1β, and IL-17 are the main cytokines linked to this inflammation. These cytokines increase the inflammatory response by drawing on neutrophils and monocytes. In the liver, IL-6 also stimulates the production of CRP and other acute-phase proteins. A threefold increased risk of postoperative complications and a longer duration of stay are associated with elevated IL-6 levels on the first postoperative day [12]. Dehydration during and after surgery might result from prolonged fasting, which increases the risk of postoperative vomiting. Preoperative carbohydrate drinks reduce xerostomia, hunger, and thirst while reducing inflammation [13] But, IL-6 and CRP levels at the beginning of the study were seen to be higher in the control group than in the intervention group due to the presence of subjects with malignancy in the control group. This may lead to biased results, further research confirmation is needed.

This study also attempted to analyze the effect of administering a combination of protein and carbohydrate solutions on IL-6 levels based on the level of surgical complexity. The results showed a significant difference in pre-operative, 24-hour post-operative IL-6 levels, and the pre-postoperative mean delta between the two groups at surgical complexity level 2. Meanwhile, at level 3, there was no significant difference in pre-operative, 24-hour post-operative IL-6 levels, and the pre-postoperative mean delta. When multivariate analysis was performed, none of the variables affected IL-6 levels, Surgical complexity level 2 resulted in a significant difference in IL-6 levels between the two groups, according to bivariate testing, although this did not occur at level 3. The degree of surgical complexity can aid in determining the risk of post-operative infection, according to Furlanetto et al. (2023). An elevated inflammatory response may result from an infection, which is more likely to occur with more difficult surgeries [14]. Consequently, there was no discernible change in IL-6 levels between the two groups in participants with surgical complexity level 3.

The results of the study showed that the control group's average CRP levels were higher than those of the group that received protein and carbohydrates, although the difference was not statistically significant. This is consistent with the study of Yi et al. (2020), which found no significant change in CRP levels between the group that received protein and carbohydrates and the control group (p=0.050) [15]. This contrasts with the study by Andreanto et al. (2015), which demonstrated a significant difference (p=0.001) in post-operative CRP levels between the control group and the group that loaded protein and carbohydrates [16]. Surgery can increase CRP. When surgery occurs, tissue damage triggers stress, which increases cytokine production, one of which is IL-6. IL-6 triggers CRP production, leading to an increase in CRP post-surgery [12]. The degree of the CRP-mediated inflammatory response has a direct bearing on this recovery. Within 4 to 48 hours following surgery, serum CRP values rise noticeably, and this rise is correlated with the degree of surgery [17]

There is much debate over how carbs affect inflammatory processes. Despite their undeniable role in insulin secretion and fat formation, there is no solid evidence linking them to direct inflammatory effects [18,19]. The surgical stress response also increases protein catabolism, which can ultimately delay wound healing and impair immune function. Administering a combination of protein and carbohydrate solutions increases protein levels, which can aid wound healing and immune function [20,21]. Additionally, preoperative fasting results in headaches, dehydration (which might obstruct venous access), anxiety, pain, malaise, increased hunger and thirst, and a delayed recovery from surgery because of an elevated stress reaction. Therefore, administering a combination of protein and carbohydrate solutions can reduce the stress response, thus lowering postoperative CRP [22]. However, an inflammatory reaction is still necessary during the healing process, occurring in the early stages. Both the innate and adaptive immune systems are activated when inflammatory cells infiltrate the body, causing inflammation. Significant pathological changes, including scarring and poor wound healing, can be brought on by excessive inflammation. On the other hand, effectively controlled inflammation usually promotes wound healing [23].

The insignificant results in this study may be influenced by several factors. CRP begins to increase within 12–24 hours of the inflammatory response and peaks at 48–72 hours [24]. This may have contributed to the insignificant results, as this study was conducted 24 hours post-surgery, when CRP levels are still increasing for 48–72 hours. Other factors include ASA status and surgical stage. In ASA 2, patients have mild systemic disease, which increases inflammation. Hypertension, diabetes mellitus, and obesity can increase the release of cytokines and ROS due to the infiltration of innate and adaptive immune system cells into the kidneys, blood vessel walls, and surrounding areas [25,26]. This study analyzed data separated by surgical complexity, at level 2, and the results showed a significant difference in CRP levels 24 hours postoperatively and the mean delta pre-postoperatively in both groups; while at level 3 this was not the case. This may be because at level 3 surgical complexity is a moderately invasive procedure, which can increase higher inflammation compared to level 2. The higher the level of surgical complexity, the greater need for a greater level of inflammation to assist in the wound healing process [27]. Even though a combination of protein and carbohydrate solutions was given, the inflammatory response remained high and caused the results of this study to be insignificant.

The limitations of this study are: 1) This study only consists of subjects who underwent surgery with surgical complexity levels 2 and 3, these results cannot represent subjects with surgical complexity levels 4, 5 and 6. 2) This study only consists of ASA 1 and 2, so it cannot represent subjects with ASA 3, and others; and 3) This study did not exclude patients with malignancies and autoimmune diseases, or those undergoing immunosuppressive therapy. There were no autoimmune patients in this study; however, one patient had a malignancy, which could have confounded the results. However, the study can sufficiently describe the relationship between the administration of a combination of protein and carbohydrate solutions to CRP, IL-6 and NLR in patients undergoing surgery because, most patients undergoing surgery are with ASA 1 and 2, with surgical complexity levels 2 and 3.

## Conclusion

A combination of protein and carbohydrate solutions can help suppress postoperative NLR, IL-6 and CRP levels, although the difference between preoperative and 24 hours postoperative was found to be significant only in IL-6 levels.
